# Healthcare professionals’ perspectives of patient and family preferences of patient place of death: a qualitative study

**DOI:** 10.1186/s12904-021-00842-y

**Published:** 2021-09-20

**Authors:** Manjusha K. Sathiananthan, Gregory B. Crawford, Jaklin Eliott

**Affiliations:** 1 The University of Adelaide, Adelaide, Australia; 2 Northern Adelaide Palliative Services, Northern Adelaide Local Health Network, Adelaide, South Australia; 3 Adelaide Medical School, Faculty of Health and Medical Sciences, The University of Adelaide, Adelaide, Australia; 4 School of Public Health, Faculty of Health and Medical Sciences, The University of Adelaide, Adelaide, Australia

**Keywords:** Palliative care, Place of death, Healthcare professionals, Home death

## Abstract

**Background:**

Home death is one of the key performance indicators of the quality of palliative care service delivery. Such a measure has direct implications on everyone involved at the end of life of a dying patient, including a patient’s carers and healthcare professionals. There are no studies that focus on the views of the team of integrated inpatient and community palliative care service staff on the issue of preference of place of death of their patients. This study addresses that gap.

**Methods:**

Thirty-eight participants from five disciplines in two South Australian (SA) public hospitals working within a multidisciplinary inpatient and community integrated specialist palliative care service, participated in audio-recorded focus groups and one-on-one interviews. Data were transcribed and thematically analysed.

**Results:**

Two major and five minor themes were identified. The first theme focused on the *role of healthcare professionals in decisions regarding place of death*, and consisted of two minor themes, that healthcare professionals act to: a) mediate conversations between patient and carer; and b) adjust expectations and facilitate informed choice. The second theme, *healthcare professionals’ perspectives on the preference of place of death*, comprised three minor themes, identifying: a) the characteristics of the preferred place of death; b) home as a romanticised place of death; and c) the implications of idealising home death.

**Conclusion:**

Healthcare professionals support and actively influence the decision-making of patients and family regarding preference of place of death whilst acting to protect the relationship between the patient and their family/carer. Further, according to healthcare professionals, home is neither always the most preferred nor the ideal place for death. Therefore, branding home death as the ideal and hospital death as a failure sets up families/carers to feel guilty if a home death is not achieved and undermines the need for and appropriateness of death in institutionalised settings.

**Supplementary Information:**

The online version contains supplementary material available at 10.1186/s12904-021-00842-y.

## Background

Many studies identify home as the most commonly preferred place of death across the general population, regardless of location or illness [1–5]. However, a systematic review of 210 studies from 33 countries found that, although home is the most commonly preferred place of death, preferences varied significantly among different cohorts: 49–70% for the general public, 31–87% for patients, and 25–64% according to caregivers [6]. Of studies examining patient preferences, most have focused on those diagnosed with cancer, with a recent systematic review reporting that their preferences for home death varied widely, ranging from 39.7 to 100% [7]. A further systematic review of 61 articles assessing adult UK preferences for place of death reported that ‘“missing data,” the views of those whose preferences were not asked, expressed or reported or absent in studies … were common.’ When adjusted for them, the authors concluded it was not possible to gauge the proportion of patient preferences for a home death [8]. A commonly-cited South Australian (SA) 2006 study surveying 2652 participants across the general population reported that 70% expressed a preference to die at home if dying of a terminal illness [9]. However, when weighed against the age-sex distribution of actual deaths of cancer patients during 2000–2002 in SA, the authors reported that the 70% statistic for the preference of a home death dropped to 58%, while 42% preferred death at hospital, hospice, or nursing home [9]. In addition, the findings of such studies may not be representative of the views of patients suffering from other terminal illnesses, as highly person-centred factors influence preferences for place of death [10].

Some studies indicate potential negative impacts of achieving home death, for example, the burden on the caregiver [10, 11]. According to some, a preferred place of death could be anywhere that the patient and their caregivers/family feel to be a ‘safe place’, has characteristics of ‘at-homeness’, and is aligned with the cultural, religious, and personal values of the dying person [3, 10, 12–14]. Hence, while home may be ‘a’ preferred place of death, claiming that it is ‘the’ preferred place, is misleading [10, 15].

Despite inconclusive evidence regarding preferences for place of death, home death is generally equated to a ‘good death’ [16]. Therefore, there has been increased attention on achieving home deaths. For example, although most recent statistics from SA (the site for the present study) show that only 38.4% of all deaths occurred in the community [17], in 2016, achieving 45–55% of deaths at home was a target goal of SA palliative care services [18]. Given this, deaths in medical settings may be perceived as a failure on the part of the healthcare system and for those working within it.

Most literature on the preference of place of death has focused on the perspectives of patients, carers, or the general population, rarely on that of healthcare professionals (HPs) [19]. However, some research suggests both that the views of the general population and those of HPs differ [20], and that the values and views of HPs have a significant influence on end-of-life decision-making, independent of variation in disease characteristics and prognosis [21]. In Australia (the setting for this study), HPs have been noted to be critical actors who significantly influenced decisions made about where a patient died [22]. This study aims to assess the perspectives of HPs across diverse disciplines within a multidisciplinary inpatient and community integrated specialist palliative care service, regarding the preference for place of death of their patients receiving palliative care.

## Methodology

A pragmatist approach underpinned by symbolic interactionism is adopted. This enables consideration of how the biological realities of death and dying and the social interpretations of these, are shaped through social interactions (e.g., with HPs), which in turn may influence preferences of place of death [23–25].

## Methods

### Sampling

Purposive sampling was conducted to recruit participants from diverse disciplines who could provide focused, rich, and relevant data on the research question [26, 27]. Forty-two HPs working with adult patients receiving end-of-life care with palliative units within two tertiary public hospitals in Adelaide, SA were informed by GBC about the study and invited to contact MKS via email if interested in participating. Four declined due to conflicting clinical commitments. Participants included 38 HPs across five disciplines: palliative care specialist doctors and doctors in training (D), nurses (N), allied health workers (AHW), social workers (SW) and chaplains (C). All were able to understand and speak English. (See Table 1 for demographic details).

**Table 1:** Participant details

Discipline	Participant N	Age range (years)	Mean age (years)	Range of years in palliative care service	Average years in palliative care service
Nurses	12	28–62	57	1–40	18
Specialist doctors	4	45–67	53	7–20	14.25
Doctors in training	2	25–26	25	0.25–1	0.62
Social workers	4	35–62	48	2–15	7.5
Allied health workers	8	30–56	46	1–10	4.5
Chaplains	8	45–75	63	2 – 30^a^	13^a^

^a^ Work experience not limited to palliative care service since the role of a chaplain routinely involves end-of-life conversations with patients and families

### Data collection and analysis

Focus groups (FG) were the primary mode of data collection (*n* = 7), as they facilitate group interaction, encouraging respondents to explore and clarify individual and shared perspectives [28]. Where key participants were not able to attend the FG meetings, one-on-one interviews (OI) were conducted (*n* = 2). After providing written consent, each participant participated in either a FG discussion (with 2–8 participants) or a one-on-one interview lasting 60–75 min. All sessions were conducted in a private meeting room in the hospitals, and facilitated by JE, supported by MKS, who also took notes. Open-ended questions were guided by a semi-structured topic list (see Additional file 1 for interview guide) based on a scoping review of the relevant literature.

Data saturation was attained by the sixth FG, but an additional FG and two interviews were conducted to include the views of HPs from all disciplines within the palliative care service. All discussions were audio-recorded and transcribed verbatim. Transcription was undertaken by MKS and a contracted company who signed standard confidentiality clauses. Initial coding of the data was conducted by MKS facilitated using NVivo software [29]. Codes were discussed, and themes derived amongst the research team [29]. Differences in interpretation were resolved through discussions, and if necessary, by referring to the transcripts (See Table 2). Participants were sent and invited to comment on the findings to improve rigour. Only one HP responded, with no corrections. (See Additional file 2 for thematic analysis summary).

**Table 2:** Steps in thematic analysis [29]

**1. Familiarise with the data** The transcripts were checked against the recordings, read and re-read by MKS, and reviewed by GBC	
**2. Generate initial codes** The codes were assigned to concepts relating to the research question	
**3. Search for themes** The codes were categorised so that themes emerged from the data	
**4. Review themes** The themes were reviewed with the research team	
**5. Define and name themes** The themes were named, and the relation between the themes was established	
**6. Produce the report** The themes were elaborated and presented in the form of a report	

### Ethical considerations

All methods were carried out in accordance with relevant guidelines and regulations.

As all participants were professionals working in palliative care, the risk of the topic evoking some emotional responses was identified as minimal. The same negligible risk applied to the researchers involved. Participant data were de-identified at transcription; however, participants were advised in writing and verbally that the use of information on their role responsibilities (e.g., doctor, nurse) and the specific locations of the study might compromise confidentiality.

We acknowledge that the researchers had some professional and/or personal experience of the topic, requiring open reflection within the research team on how this might shape the research process. The first author (MKS) is a healthcare professional and aged care worker. As a teen, she witnessed the home death of her grandfather, which she remembers as a good death. GBC is an experienced palliative care specialist doctor with current working relationships with participants, thus was neither present during data collection nor had access to data before de-identification. Finally, JE is a qualitative researcher with extensive experience in working with patients, families, and healthcare professionals on end-of-life issues.

## Results

Two major themes were identified from the data:

- Theme 1: The *Role of healthcare professionals in decisions regarding place of death* is comprised of two sub-themes: to a) mediate conversations and protect relationships between the patient and their carers; and b) adjust expectations and facilitate informed choice.
- Theme 2: The *Healthcare professionals’ perceptions of the preference for place of death* includes three sub-themes: a) characteristics of the preferred place of death; b) home as a romanticised place of death; and c) the implications of idealising home death.

### The role of healthcare professionals in the decision-making process

#### To mediate conversations and protect relationships between the patient and their carers

This theme was commonly expressed by doctors, nurses, and allied healthcare workers, but rarely by chaplains and social workers. Across interviews, many HPs observed that a lack of open conversation regarding the place of death between the patient and their caregivers. They added that this failure to communicate openly is further exacerbated when the preferences of patient and carer do not coincide. Some HPs recognised that when care needs are high, as is common, there is a ‘loss of identity’ of carers, who often feel ‘confronted’ and ‘distressed’ due to an unanticipated change in their relationship with the patient: explicitly, that the carer role often superseded their role as a family member. Furthermore, all HPs noted that when the demand at the end of a patient’s life becomes ‘overwhelming’ and ‘exhausting’, carers sometimes recognise their inability to cope with the stress of caregiving and/or the anticipated trauma of the death of their loved ones at home, and thus, may ‘surrender’ and change their minds about wanting a home death for the dying person. A doctor stated:

The change [of the preference] over time, [due to] absolute exhaustion, we see that “I can’t do this anymore”. (D/FG)

Some HPs noted that in some cases, ‘the promise’ made by the carer to the patient to care for them at home until the end or, in many cases, the implicit expectation to do so, often constrained a carer’s expressions of their change in preference. Many HPs observed that in those cases, carers feel ‘immensely guilty’ and that it is confronting for them to express their inability to keep this promise and thus deny the patient’s wish to die at home. Therefore, some HPs stated that, to avoid hurting the patient and/or damaging carers’ relationships with them, carers often refrain from voicing such concerns to the patient. For example, a doctor quoted a carer as stating:

“I promise I won’t send you to nursing care [residential aged care home], I’ll care for you at home,” and then the care needs are too great. And the guilt and the suffering of the person who is unable to make good on their promise can break them. It’s really significant. (D/FG)

In such situations, according to some HPs, carers often resorted to disclosing their predicament to the HP when alone, without the patient. Many HPs relayed that, sometimes during home visits, carers followed them outside to their car or met them at the letterbox to express their concerns privately. All HPs considered that, although often the carers cannot or do not ‘speak-up,’ it is crucial to elicit carers’ opinion regarding the patient’s preference of home death. Some HPs, therefore, stressed the need to have ‘dual conversations.’ Many HPs saw it as part of their duties to listen to carer problems and acknowledge their struggle. However, they found it ‘tricky’ to have such conversations without the knowledge of the patient and maintain confidentiality. Some spoke of reassuring the carer that they are not ‘failing’ for not being able to cope with the expectations of caregiving at home. For example, a nurse characterised their interaction with carers in such cases as:

It’s that reassurance that – “it’s OK that you can’t look after them. They are taken care of properly in hospitals and nursing homes.” There are people around that can help 24/7, rather than at home [where] it’s only them. (N/FG)

In addition, to help maintain the relationship between the patient and the carer, HPs commonly spoke of taking on the role of the ‘baddie’ who delivers the bad news to the patient that their preference for home death may not be feasible. For example, an allied health worker explained:

If you have a had a conversation with a family member, they don’t want to ruin that relationship they’ve got with the patient, over … not being able to voice that they can’t care for them at home, and emotions that surround that, but sometimes we have to be the baddie to give that news to maintain that sort of relationship. (AHW/FG)

#### To adjust expectations and facilitate informed choice

In order to promote informed decision-making of the patient-family unit, according to many HPs, it is important to determine the patient-family unit’s understanding and expectations regarding the end of life. Some HPs observed that, generally, Australian society is ‘death-denying’ and ‘ill-prepared’ to deal with death and dying. They further claimed that this typically sees the patient-family unit with a poor understanding regarding the realities and consequences of caring for someone at the end of life, often resulting in making commitments like ‘the promise’. Furthermore, most HPs identified that the uncertainties, fluidity, and highly emotional nature of circumstances at the end of life make it difficult for the patient-family unit to arrive at a deliberated decision regarding the place of death. Under such circumstances, many HPs claimed that patients and family often rely on their guidance to identify realistic options available and thus enable them to make the ‘right’ choice. Some HPs further acknowledged that their judgements regarding the best place of death for their patient often influence the care plan for their patients from the outset. A doctor described the need for informing choices as:

I suppose there’s really the fact that people so rarely see death and so rarely see how someone changes as they deteriorate and how, you know, what care needs someone has. So, from the beginning when these promises were made, they don’t know how things will look, and it’s part of our role, that we’ve seen it, that we can actually help guide family and patients so that we’re doing it in a supportive way, but really, we’re guiding them in terms of what we’ve seen is going on and say, “you know, we can try and put in as much services as we can, but really, your condition is such that it would be just too hard for you to be at home”. So, I suppose, you know, really, giving them that knowledge that they didn’t have at the beginning when, as I said, these promises were made. (D/FG)

The same doctor elaborated on how he ‘frames’ the issue when informing realistic choice:

“I wish we could let you honour that promise, but [we] cannot provide enough support to do it.” And that gives permission for the death in hospital, whereas a different approach may drive much harder to going home in an untenable circumstance. (D/FG)

All HPs identified that their influence on the preference of place of death begins with their assessment of the circumstances specific to the particular patient-carer/family unit. Following this, they make judgments on realistic options for place of death available to each patient—especially considering the likelihood of achieving a home death, if that is preferred. They noted three primary factors they considered in making this assessment. First, the characteristics associated with the disease of the patient, including the care needs consequential upon a projected illness trajectory. For example, as observed by some HPs, end-of-life presentations of some diseases may be traumatic for the family, especially for young children. In such cases, HPs noted that they sometimes advised against aiming for a home death. One social worker recalled such a traumatic death at home as:

We had a fellow who discharged himself to die at his niece’s place where he had been staying for the last few weeks with her as his carer, and he came home, and he died two hours later with a catastrophic bleed; he bled out in her arms. And this is traumatic for anyone to witness, and no doubt it had been flagged because there are certain cancers that, cancers have good blood supply and certain ones are likely to come out, if it’s a throat cancer for instance, [with] a lot of blood. And that person had flashbacks and all kinds of trauma from it. (SW/FG)

Some participants stated that despite a strong preference to stay at home, caring for a patient, especially where they were obese, at home may be impractical, thus engendering a ‘need’ to transfer to a hospital.

A second factor considered was the availability, willingness, and ability of the carer to provide basic and medical care for potentially ‘23 hours of the day’. Some HPs pointed out that according to the current model of care, most people at their end of life receive only an hour of service in a day from professional care workers, which makes the participation of informal carers crucial. Without active carer participation, all HPs considered a home death both unlikely and not ideal. According to most, the common reasons for not meeting this requirement are when the patient lives alone, and/or when the carer has physical limitations (e.g., frailty) or work commitments, is unable to cope with the emotional demands of providing care or is unwilling to shoulder caregiving responsibilities due to strained relations with the patient.

The final circumstances mentioned included the patient’s priorities and the specific nature of the patient-family relationships. Here, many HPs stated that they considered the personal relationships (within the family, vertically and horizontally), as well as the patient’s values and beliefs shared (though not always explicitly) within the family unit. HPs observed that these factors rendered the process and decisions as highly variable and unique. For example, many identified that, for some patients, the provision of adequate and timely symptom control weighed more than the desire to die in a preferred location; for others, being with their pets at the end of life took priority. Some added that, in some families, the cultural and spiritual beliefs that prioritised achieving a home death might prevail over any personal preferences. Ultimately, all HPs noted that the judgment about the best place to die varies for each patient. An allied health worker explained this as:

I think it does vary. I mean if people have confidence in their family or they have this emotional, very deep emotional attachment to their home, they see that the value is in being in that space. But then there’s the flip side of those that have uncontrolled nausea or vomiting or pain, and they don’t want to be at home, they’re scared to be home, and they’ve become dependent on being in the [palliative care] unit especially as things get worse towards the end, … or if is it culturally more appropriate for them to be at home for the end of life. And that’s something that we need to consider no matter what is going on with the patient. (AHW/FG)

### Healthcare professionals’ perspectives on the preference of place of death

According to some HPs, it is often difficult to elicit the preference of place of death due to the unwillingness of patients to have a conversation about their death, especially when they are not in the terminal phase. Therefore, some HPs stated that they often alluded to the issue of the place of death and recorded an implied answer. They noted, however, that this practice often leads to a weak distinction between the preference for place of care and for the place of death, posing a barrier to identifying an actual choice of place of death of patients. A doctor remarked:

We don’t often ask, we don’t often differentiate the two, but I think it’s really important, like, “where do you want to be cared for, where do you want to die?” I think they could be two separate answers. (D/FG)

Many HPs reported that people commonly express a preference to stay at home as long as possible, often conditional on the presence of features discussed below.

#### Characteristics of a preferred place of death

According to HPs, although some patients expressed an unwavering preference to die in their own space in their own home, for most, it was not simply a preference for a physical space that was important at the end of their life. Rather, it was particular characteristics of that space. For example, participants in all interviews noted that dying patients typically want to have a home death to be close to their family and pets at the end of their life. However, most observed that home is not the only location which might possess the most desired characteristics of an ideal place of death; these can be created at any location. For example, a social worker remarked:

And that question of what home actually is and what does that mean is, are there ways of being able to achieve that in a different location for people, because often home is people, and that sometimes can be achieved in other ways. (SW/FG)

However, they additionally observed that patients often are strongly motivated to protect their loved ones from the burden of caregiving and a predicted trauma of their death at home. In such circumstances, some HPs note that an institutionalised setting, typically a palliative care unit, is often preferred for the place of death. According to some HPs, such an option of a place of death, where there is provision for the family to stay and bring in pets while ‘outsourcing’ care and death, is often acceptable for patients, leaving home as a ‘good place’ or ‘respite’ for the family. A chaplain quoted a patient as saying:

“OK, when I’m about to die, you can transfer me to the hospital, so I am not at home here so that the place becomes a good place, in a different way, for my wife.” (C/FG)

Many HPs noted the importance of a ‘home-like’ environment of a place of death for patients. They observed that a nursing home or a hospice which is more ‘home-like’ tend to be favoured by patients as a place of death rather than those with a ‘clinical style’ setting. Some HPs observed that patients often valued the opportunity to personalise one’s surroundings. Furthermore, according to many HPs, familiarity with space was a valued characteristic and often the reason for choosing home as place of death. They noted that, in addition to a preference for familiarity with the physical things like one’s ‘own bed’ and ‘comfortable chair’, familiarity in the sense of ‘memories’, ‘routines’, knowing and ‘trusting’ the people around them is important for patients. For example, according to some HPs, a nursing home may be a preferred place of death where patients reside there for a long time, ‘build relationships’ with people around, and the staff ‘feel some connection’ with them. Similarly, a few HPs observed that if a patient has spent much time in hospitals (perhaps for treatment of chronic illness), and is familiar with that setting, they are more likely to name the hospital as the preferred place of death. A doctor summed it up as:

I think people want to be in their own familiar surroundings with all the psychosocial factors associated with that. (D/OI)

Many participants identified safety as another aspect of a setting which made it preferable as a place of death. Home, being known, is often identified as the ‘safe place’; however, many HPs noted that at the end of life, home may not always be the physically safest place for patients, especially for those with frailty. Moreover, many HPs opined that, as most dying patients experience an increasing symptom burden towards the end of life, requiring higher levels of care that families may be ill-equipped to provide (either emotionally or practically), as death approached, home may not be a safe place either for patient or family. In such cases, a hospital provides safety, as noted by a nurse:

They can come to a point where they feel safe in a hospital. They can get a bit anxious at the thought of going home. (N/FG)

Many HPs also observed that patients highly valued the ability to exercise autonomy at the end of life. Here, the home would be named as an ideal setting, partly because patients and families were able to have maximum freedom. Conversely, the hospital was not, partly because of the institutional rules and restrictions. However, a doctor pointed out that sometimes hospital restrictions are appreciated by patients:

Sometimes coming into hospital can be easier because there’s not all the visitors always dropping in…. When people are sick and unwell and tired, they love seeing people, but some people outstay their welcome, you know. They stay too long, and they are exhausting, and we see that even in the hospice or palliative care unit, that they will come and there may come a time to restrict the number of visitors and the duration of visits. (D/OI)

According to all HPs, both having and knowing that a patient’s care needs will be met factor into a preference for place of death. Most HPs identified that, for patients with high care needs or where the carer is unable or unwilling to care for the patient, institutionalised settings become the only realistic option and hence are accepted as the practical choice of place of death. They further added that sometimes such a setting is preferred as it can best provide responsive symptom control and meet the care needs of patients. As noted by a nurse:

Because they’re comfortable in [the] hospital, that they know they can ring the bell, the nurse will come, the problem is fixed. Whereas at home, [the] partner might have gone down the shops, “I’ve had some pain. I’ve got to wait. How long is he going to be?” (N/FG)

Contrarily, another nurse observed that sometimes carers feel that they could have provided more responsive care at home, raising the issue of hospitals being understaffed. She quoted a carer as saying:

“I wish she would be at home when she is dying because I could have been more responsive. I had to wait for the nursing staff.” So, I think acute hospitals these days are often understaffed around [for] significant duration. (N/FG)

Finally, some HPs noted that preferred care involves maintaining the dignity of the patient. For example, they observed that a real or anticipated loss of independence at home and increased dependency on a familial carer to meet personal and intimate care needs (e.g., toileting) might prompt a shift in preference from home to the hospital. A nurse explained it as:

Sometimes people will say, “I never want my children to look after me if I’m incontinent.” So that’s a bit of a line in the sand for people. (N/FG)

#### Home as a romanticised place of death

Participants within all groups observed that preferences for home death were typically predicated on a romanticised notion of death and dying, invariably obtained from movie portrayals, rather than from an informed understanding of the physical, emotional, and clinical realities of death and dying. As noted by a social worker:

… often dying at home is romanticised, and it’s not a realistic understanding of what it would be, how it’s meant to be like a Hollywood movie, and there’ll be some perfect resolution of all of this distress and everything around it in this perfect moment. It often isn’t. (SW/FG)

#### Implications of idealising home death

A few HPs raised concerns that idealising a death at home has adverse consequences for the carer if they are unable to accommodate this. A social worker noted:

But the challenge with the measure of home versus not at home, for those families that don’t achieve home, then that’s a failure. It’s a huge, huge failure, … they go, “I shouldn’t have called the ambulance. I shouldn’t have got them taken to hospital. I promised. I did this; I did that.” That’s a horrible outcome. (SW/FG)

Many HPs contested the appropriateness of home death as a quality measure for palliative care service, some asserting that death at a hospital could be a ‘good death.’ One doctor said:

I dislike the feeling that death in hospital is a failure of the system, particularly when that can be somebody’s preference. … So, if we’ve kept someone out of [the] hospital for 99 days and they’ve come into [the] hospital for that 100^th^ day when they die, then I think that is superb palliative care if that was their wish. (D/FG)

## Discussions

Some studies have explored HPs views and responsibilities toward the patient and their family with regards to the general end-of-life issues [30, 31], but this study is focused on the topic of place of death from the perspectives of a team of integrated inpatient and community palliative care service staff. This study analyses HPs’ views of their involvement in the decision-making process of the preferences for place of death of their patients, and the meaning behind patients/carer preferences for place of death of patients. It uniquely describes the nature of HPs engagement with the family unit in the choice of place of death of patients—by mediating conversations between the patient and their carer, adjusting expectations and facilitating informed decision-making, whilst acting to maintain a positive relationship between patient and carer. According to HPs in this study, home is not invariably the most preferred place of death and the assumption that it is has negative consequences for patients, the carers, and the HPs themselves.

HPs’ perspective on the problems faced by carers adds to the existing knowledge on the issue of the burden of caregiving at the end of life [32–34]. Similar to the present findings, the carers’ situation at the end of life has been described previously by HPs as ‘struggling with helplessness’ and ‘having unspoken and conflicting emotions’ [35]. This study adds a nuanced understanding of the carer experience from HPs’ perspective within the context of decision-making regarding the place of death. Carers’ struggle to cope with the demands of caregiving in order to keep ‘the promise’ made to the dying patient was consistently observed by the HPs, further alluding to the moral distress and guilt experienced by carers caught between the romanticised and real versions of dying. This finding is consistent with reports that carers are less likely to prefer a home death than patients, and in some cases, where home death is achieved, carers’ retrospective judgement that hospital would have been a better place of death [6, 36].

These results support findings that the determinants of a home death, as observed by HPs, include factors such as the patient’s disease, mobility, and physical size; family support, and availability of facilities and services [30]. The significance of personal values and culture on the choice of place of death are previously reported [2, 10, 37]. HPs’ awareness, however, of their influence on place of death has not been recorded before.

Many studies have noted temporal variability of preference of place of death [1, 10, 38], with the preference for home death decreasing and that for hospital death increasing with time (or more accurately, as the illness progresses) [39]. This study provides a more elaborate account of the nature and consequences of such changes in the preference, noting the influential role of HPs in supporting carers where their preferences had changed from home to hospital, typically following an increase in patient symptoms and carer burden. HPs saw themselves as morally bound to claim decisional authority, protecting patient and carers, as well as the relationships between them. This finding echo that of a 2003 Australian study which noted HPs as navigating complex situations when striving to achieve a good death for the patients, including taking some control of the decision-making at the end of life. Nonetheless, this was in the context of HP’s attempts to maximize patient autonomy and to avoid involvement in family dynamics [40]. In contrast, our study finds that HPs feel responsibility and concerns for familial relationships, even where the choice of the family subverts the patient’s preference. Another Australian study reported that, amongst Chinese communities the individualistic and ‘do-it-yourself’ model of end-of-life planning was inconsistent with their highly family-centred cultural approach to end-of-life decision-making [41]. The perceived valid influence of the family unit on decisions regarding the end of life constitutes a challenge to assumptions regarding an unquestioned moral (and legal) authority of patient decision-making based solely on their definitions of a ‘good death.’ It also supports the finding that blanket protocols for such end-of-life discussions is not applicable to the complex situations demanding significant interpersonal and negotiating skills that HPs working in palliative care routinely navigate [42]. However, such skills to deal with the social and relational aspects of palliative and end-of-life care are not adequately imparted to HPs during their training [43].

According to previous studies, the characteristics of a home which makes it a preferred place of death include being home-like, safe, comfortable, peaceful, and enabling the patient to be close to the family [1–3, 37]. In this study, however, HPs specifically observed that it was these *characteristics*, rather than the *location*, of the home, that most commonly determined preferences for place of death of their patients; and that such characteristics could be cultivated elsewhere. At times, dying in hospital enabled both patient’s and family’s care needs to be met, and for the home to remain ‘a safe place’ for the family. These findings support other reports that a patient’s need to protect family members is often the leading reason not to prefer home death [44, 45], but further highlight variability in reasons patients and carers might not prefer home death. Even an acute hospital setting has been noted by HPs to sometimes be considered a ‘safe haven’ for patients and their family members towards the end of the patient’s life [46]. According to HPs, this suggests a need to shift the focus from achieving home death to improving service provisions and better accommodating individual priorities at all potential places of death. This finding captures the concept of ‘placing work,’ where HPs in palliative care modify caring places to promote valued characteristics (e.g., security and familiarity), sometimes by trying to reproduce what is valued elsewhere in the chosen setting (e.g., increasing familiarity in hospital locations by personalising spaces and developing relationships with patients and family) [47].

This study supports claims that a weak distinction between the preference of place of care and that of death when eliciting patient preferences poses a challenge in recording an actual preference of place of death [19, 30]. HPs’ views that a home is often a romanticised place of death for the majority further complicates the determination of the realistic choice of place of death at the end of life. The evidence that people commonly hope for a pain-free, peaceful and quick death (as often depicted in popular media) may be the context for such romanticised preferences for home as place of death [48, 49]. However, few studies have accounted for variation over time, specifically, that patients and families may express a preference for home death as a ‘wish’ in a future yet come to recognise that this is neither feasible nor desirable at the end of life [10, 22, 50]. In a study conducted in the UK, these ‘open-ended’ and ‘unresolvable’ characteristics in the nature of ‘choice’ of place of death and the difficulty in eliciting preferences at all, combined with bureaucratic demands to fulfil organizational metrics of achieving patients’ preferences regarding place of death was found to leave HPs frustrated [47]. Similarly, whilst HPs noted the influence of the framing of the question and both patient and carer imagined version of the patient’s death, these are not considered in most studies recording preferences for place of death [6].

When healthcare policies promote home death, and idealised notions of death and dying circulate among the public the services provided to dying patients at other settings at the end of life may be compromised. A study of government palliative care policies of five countries, including Australia, found that the policies consistently ‘problematised’ hospital deaths and did not reflect the importance and need of service provision in hospitals at end-of-life [51]. Thus, based on these study findings, there is a need to affirm that death in a hospital can be a desired, appropriate, and good death for many. In addition, as the number of hospital deaths is predicted to increase into the future [52], maintaining home death as *the* societal measure of a good death seems likely to cause unwarranted guilt and distress to those families/carers who are unable to facilitate it [53]. Moreover, such standards disregard HPs significant efforts to maximise a patient’s autonomy and achieve their preferred place of death [40] —sometimes striving to create spaces, within an institutional setting, that possess desired characteristics of a preferred place of death for both patient and family [47].

### Strength and limitations

This study explores the relatively understudied opinions and perspectives of HPs regarding patient preferences of the place of their death. We acknowledge that the participants were drawn from a single palliative care service and that the circumstances of patients and families who were not referred to the service may differ. Thus, the results of this study may have limited relevance regarding the perspectives of HPs working with a different population. Nonetheless, engaging with HPs from all disciplines attending to patients referred to a palliative care service enabled a diverse and comprehensive understanding of the issue.

## Conclusions

The study found that HPs reported acting to influence decisions about place of death of patients, identified that the nomination of a home as the preferred place of death often rests on a romanticised notion of death and, when patients and families are faced with the real emotional and practical demands and care needs associated with death and dying, death in an institutionalised setting is often preferred. HPs further identified that *any* setting which facilitates being close to and protecting loved ones, that offers a safe, home-like, and familiar environment wherein patients can exercise autonomy, and where care needs of patient and family are met, may be nominated as a preferred place of death by patients at the end of their life. Therefore, HPs argued that a good death can be achieved in a hospital setting, and hence whether a home death happens or not is a poor measure of the quality of palliative care services.

Further research, including in real-time, is needed on patient and family perspectives regarding the meaning of home death and the processes by which decisions about place of death are made, shared, and change over time. Finally, based on this research, there is a need to improve individual, familial, and community awareness of the often changing realities of dying (whether at home or elsewhere); provide training to ensure that HPs are well equipped to provide support for the sometimes difficult conversations needed to attend to potentially conflicting care needs within families facing death; acknowledge the importance of institutionalised settings as a place of ‘good’ death; and, to shift the focus from promoting home deaths as default to improving the quality of palliative care services at all potential places of death.

## Supplementary Information

**Additional file 1.** Interview guide

**Additional file 2.** Thematic analysis summary

## Data Availability

The datasets used and/or analysed during the current study are available from the corresponding author on reasonable request.

## Abbreviations

HP: Healthcare professionals
FG: Focus group
O: one-on-one interviews
D: Doctors
N: Nurses
SW: Social workers
AHW: Allied health workers
C: Chaplains
