# Supplementary material for: Healthcare professionals’ perspectives of patient and family preferences of patient place of death: a qualitative study
Source: BMC Palliat Care. 2021 Sep 20;20:147. doi: 10.1186/s12904-021-00842-y (PMC8454022; doi:10.1186/s12904-021-00842-y)
Supplement: Supplementary file 1 — Additional file 1. Interview guide [file 12904_2021_842_MOESM1_ESM.docx]

**Additional file 1: Interview guide**

Question guide used for the focus group and one-on-one interviews:

1. What do you think the patients and their families consider the most preferred place to die?

•What do you think are the reasons for choosing a specific place of death?

2. In your experience, what are the characteristics of patients who prefer to die

- at home

- in hospital

- in a residential home

3. Do factors such as age, gender, family structure, religion, socio-economic status play a role? If so, why?

4. When would be the right time to talk to a patient about the preference of place of death? When do people want to talk about their preference of place of death?

5. Does the preference of place of death change with time? If so, what prompts the change?

6. In your opinion, how important is it that a patient (and their family/carers) achieves death at a preferred place?

7. What are the factors associated with achieving/not achieving death at the preferred place?

8. How common is it that the patients have a different preference in relation to a place of death than their family members? Can you please provide (non-identified) examples of how the differences in opinion are resolved?
