# Supplementary material for: Healthcare professionals’ perspectives of patient and family preferences of patient place of death: a qualitative study
Source: BMC Palliat Care. 2021 Sep 20;20:147. doi: 10.1186/s12904-021-00842-y (PMC8454022; doi:10.1186/s12904-021-00842-y)
Supplement: Supplementary file 2 — Additional file 2. Thematic analysis summary [file 12904_2021_842_MOESM2_ESM.docx]

**Additional file 2: Thematic analysis summary**

| THEMES | SUB-THEMES | CODES |
| --- | --- | --- |
| 1. ROLE OF HEALTHCARE PROFESSIONALS IN THE DECISION-MAKING PROCESS | 1.1 To mediate conversations between the patient and their carer | Carer not able to express disagreement to the patient |
|  |  | Carer stress causes a change in preference for home death |
|  |  | Carer wants HPs to be the ‘baddies’ |
|  |  | Healthcare professionals try to create space for everybody to be heard |
|  | 1.2 To facilitate informed decision making and adjust expectations. | The judgement regarding the best option is derived |
|  |  | Adjusting expectations |
| 2. HEALTHCARE PROFESSIONALS’ PERSPECTIVES ON THE PREFERENCE OF PLACE OF DEATH | 2.1 Characteristics of a preferred place of death | Autonomy |
|  |  | Being close to loved ones |
|  |  | Care needs being met. |
|  |  | Familiarity |
|  |  | Home-like environment |
|  |  | Protect family |
|  |  | Safety |
|  | 2.2 Home as a romanticised place of death | Necessary to distinguish between the place of care and place of death |
|  |  | Romanticised images of end-of-life |
|  |  | Strong preferences for home death- ‘the promise’ |
|  |  | The preference is flexible: ‘home as long as possible’ |
|  | 2.3 Implications of idealising home death | Hospital death, not a failure |
|  |  | Negative consequences to family and carers |
